# Extraembryonic mesoderm cells derived from human embryonic stem cells rely on Wnt pathway activation

**DOI:** 10.1111/cpr.13761

**Published:** 2024-10-09

**Authors:** Si‐Le Wang, Gao‐Hui Shi, Kui Duan, Yu Yin, Tianqing Li

**Affiliations:** ^1^ State Key Laboratory of Primate Biomedical Research; Institute of Primate Translational Medicine Kunming University of Science and Technology Kunming Yunnan China; ^2^ Yunnan Key Laboratory of Primate Biomedical Research Kunming Yunnan China; ^3^ Department of Anatomy, College of Preclinical Medicine Dali University Dali Yunnan China

## Abstract

Extraembryonic mesoderm cells (EXMCs) are involved in the development of multiple embryonic lineages and umbilical cord formation, where they subsequently develop into mesenchymal stem cells (MSCs). Although EXMCs can be generated from human naïve embryonic stem cells (ESCs), it is unclear whether human primed ESCs (hpESCs) can differentiate into EXMCs that subsequently produce MSCs. The present report described a three‐dimensional differentiation protocol to induce hpESCs into EXMCs by activating the Wnt pathway using CHIR99021. Single‐cell transcriptome and immunostaining analyses revealed that the EXMC characteristics were similar to those of post‐implantation embryonic EXMCs. Cell sorting was used to purify and expand the EXMCs. Importantly, these EXMCs secreted extracellular matrix proteins, including COL3A1 and differentiated into MSCs. Inconsistent with other MSC types, these MSCs exhibited a strong differentiation potential for chondrogenic and osteogenic cells and lacked adipocyte differentiation. Together, these findings provided a protocol to generate EXMCs and subsequent MSCs from hpESCs.

## Graphical abstract

Human primed embryonic stem cells drived EXMCs by activating the Wnt pathway showed EXMC characteristics and the sorted EXMCs were able to generate significant quantities of ECM proteins and E‐MSCs in the process of chondrogenesis and osteogenesis.

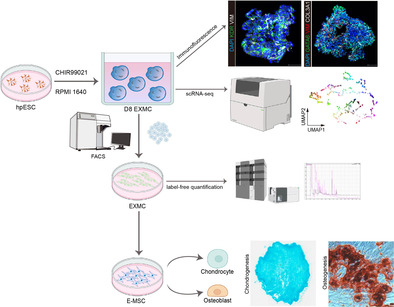

## 1 INTRODUCTION

The proliferation of human extraembryonic mesoderm cells (EXMCs) undergoes rapid expansion during embryonic development from blastocyst to gastrula, resulting in their substantial accumulation in post‐implantation embryos. This phenomenon has a pivotal role in subsequent embryonic development.^1^, ^2^, ^3^ In vitro extension culture and histological studies on human embryos show that EXMCs gradually expand between cytotrophoblast and primary yolk sac.^1^, ^4^, ^5^ EXMCs provide structural support essential for the development of various cellular lineages.^6^ In the post‐implantation embryo, the reticulate EXMC structure has a critical significance in fostering the differentiation of three germ layers, including the ectoderm, endoderm and mesoderm within the epiblast (EPI), the formation of secondary yolk sac and the expansion of the amniotic cavity, among other processes.^7^, ^8^ This anatomical structure serves as a pivotal conduit facilitating nutrient exchange between the embryo and the placenta, establishing a critical link with the maternal environment.^1^, ^9^ Due to ethical and technical limits, the development of EXMCs in a human embryo has not been fully understood. Currently, several models employing human pluripotent stem cell (hPSC)‐derived EXMCs have been established to understand the EXMC differentiation process and regulatory mechanism.^10^, ^11^, ^12^, ^13^, ^14^ Those studies focus on the developmental origin of EXMCs. However, their function still needs to be further verified. Therefore, the establishment of an in vitro EXMC differentiation model with a high purity is significant for addressing this knowledge gap.

During the in vitro EXMC differentiation, some studies have demonstrated that inhibition of the NODAL and TGF‐β pathways, coupled with concurrent activation of the mTOR and BMP pathways, facilitates the differentiation of naïve hPSCs into EXMC‐like cells and trophoblast stem cell (TSC)‐like cells in vitro.^11^, ^12^, ^14^ Similarly, hPSCs can also differentiate into EXMC‐ and TSC‐like cells after the BMP4 treatment.^10^, ^13^ The acquisition of these EXMCs in vitro goes through a primitive streak (PS) stage characterised by the emergence of TBXT (T)‐positive cells, suggesting that the EXMCs are closer to the extraembryonic mesoderm (ExM) from the post‐implantation gastrula stage.^15^ The BMP pathway is particularly crucial for naïve hPSC‐derived EXMCs in vitro. It plays a pivotal role in activating the Wnt pathway, leading to subsequent ExM differentiation.^16^, ^17^, ^18^ However, the key role of the Wnt pathway during EXMC generation in vitro remains unknown.

EXMCs exhibit prolific extracellular matrix (ECM) secretion, functioning as a reservoir for cellular resources. Notably, EXMCs demonstrate the capacity to generate a plethora of ECM proteins, especially collagen (COL1A1, COL1A2, COL3A1, COL4A1 and COL6A1), fibronectin (FN1) and laminin (LAMA1 and LAMB1), thereby establishing a conductive niche for the development of other cell lineages.^19^, ^20^, ^21^ In Carnegie stage 7, EXMCs connect the amnion, EPI disc, secondary yolk sac and cytotrophoblast to form the stalk.^22^ They subsequently migrate toward the stalk region, giving rise to the primitive umbilical cord and transforming into mesenchymal stem cells (MSCs).^13^, ^23^ The umbilical cord has extensive applications in cell therapy, particularly in generating a substantial quantity of MSCs.^24^, ^25^ However, it is unclear whether human EXMCs have the potential to secrete ECM proteins and differentiate into MSCs.

In the present study, a three‐dimensional (3D) differentiation procedure was developed to generate EXMCs in vitro. Human primed ESCs (hpESCs) were subjected to a 48‐h treatment with the Wnt pathway activator CHIR99021 (CHIR), resulting in the generation of a large number of EXMCs during the 8‐day differentiation period. Single‐cell RNA‐Seq (scRNA‐Seq) analysis and protein detection showed that hpESC‐derived EXMCs closely resembled embryonic EXMCs in terms of the gene expression pattern. The label‐free quantitative proteomics (Label‐free) analysis showed that these EXMCs produced more alpha 1 chain of type III collagen (COL3A1) compared to umbilical cord MSCs (UC‐MSCs). Furthermore, EXMCs demonstrated the potential to differentiate into MSCs. Therefore, the hpESC‐derived EXMC model provided a new idea for the application of EXMCs.

## 2 MATERIALS AND METHODS

### 2.1 Cell lines

Two hpESC lines h1 and h2 used in the present study were isolated and cryopreserved.^26^, ^27^ UC‐MSCs were isolated from Wharton's jelly.^28^, ^29^

### 2.2 Human ESC culture

The hpESC lines h1 and h2 were cultured in AIC culture medium developed as previously described.^26^ The AIC medium consisted of modified N2B27, 10 ng/mL Activin A (Peprotech, 120‐14E, USA), 2 μM IWP‐2 (Selleck, S7085, Houston, TX, USA) and 0.6 μM CHIR99021 (Selleck, S2924). The hpESCs cultured on a plate precoated with Matrigel (Corning, 354,277, NY, USA) were washed twice with phosphate‐buffered saline (PBS), treated with 50% TrypLE (Gibco, 12,605,028, Gaithersburg, MD, USA) for 3 min, collected and centrifuged (1000 r/min for 5 min at room temperature) in PBS, resuspended in the AIC medium with 2 μM Y‐27632 (Sellck, S1049) and seeded on a new Matrigel‐coated plate. Cells were routinely passaged at a 1:10 split ratio every 3–4 days. The medium with 2 μM Y‐27632 was changed every 2 days.

### 2.3 EXMC differentiation

After reaching 80%–90% confluence, hpESCs were dissociated into single cells using TrypLE for 5 min. On the starting date (day −1, D‐1) of the differentiation experiment, 20,000 hpESCs were resuspended in the AIC medium containing 10 μM Y27632 and seeded on AggreWell plates (AggreWell™400, Stemcell technologies, 34,411, Vancouver, BC, Canada) for 24 h to enable the generation of cell aggregates. The AIC medium was replaced on day 0 (D0) with the EXMC medium, which consisted of Advanced RPMI 1640 medium (Gibco, 12,633,012), 2% B‐27 Supplement XenoFree Minus Insulin (Gibco, A3695201), 10 μM Y‐27632 and 2 mM GlutaMax (Gibco, 35,050–038). Three different time courses were evaluated for CHIR99021 (CHIR) (3 μM, Selleck, S2924) in order to verify the function of CHIR for promoting EXMC formation, including CHIR‐24 h, CHIR‐36 h, CHIR‐48 h and CHIR‐72 h. Cells were cultured with CHIR for 24 h, 36 h, 48 h and 72 h to allow the aggregated cells to form embryoid bodies (EBs). Then, the CHIR in the EXMC medium was removed according to the time course. The EBs were transferred to the Ultra‐Low Attachment six‐well plates (Corning, CLS3471‐24EA) on day 3 (D3) and cultured in the EXMC medium until day 7 (D7).

To validate the key role of the Wnt pathway in promoting the EXMC generation, the Wnt inhibitors were added following the same cell culture process as describe above. On D0, 1 μM BIO (Selleck, S7198), 2 μM IWP‐2 and 2 μM XAV‐939 (Selleck, S1180) were added to the EXMC medium for 48 h. On D2, the BIO, IWP‐2 and XAV939 were removed from the EXMC medium. On D3, the EBs were transferred to the Ultra‐Low Attachment six‐well plates and cultured in the EXMC medium until D7. The culture medium was changed every 2 days. Cells were incubated at 37°C in 5% CO_2_. Cell images were acquired using DMIL LED Leica (LAS v4.9, Wetzlar, Germany).

### 2.4 Flow cytometry and cell sorting

The hpESC‐derived EBs were dissociated into single cells using TrypLE for 5 min. Then, the cells were washed three times with 2% foetal bovine serum (FBS; Gibco, 10099141C)/PBS at room temperature, resuspended in 100 μL of 2% FBS/PBS and incubated with the primary antibodies on ice for 30 min in the dark. After washing three times with 2% FBS/PBS, the secondary antibody (donkey anti‐Goat IgG (H + L) Cross‐Adsorbed Antibody, Alexa Fluor™ 647, Carlsbad, CA, Invitrogen, A‐21447) was added and incubated on ice for 30 mins in the dark. After washing three times with 2% FBS/PBS at room temperature, the cells were resuspended in PBS and placed on ice. Flow cytometry analysis and cell sorting were performed using the BD LSR Fortessa (BD Biosciences, San Jose, CA, USA) or the BD FACSAria SORP (BD Biosciences). The data were analysed using FlowJo software (version.10, BD Biosciences). The primary anti‐LUM antibodies (rat IgG1APC isotype control; Table S1) were used for EXMC^LUM+^ sorting. The anti‐BST2 antibodies (mouse IgG1PE isotype control; Table S1) were utilised for EXMC^BST2+^ sorting. Other antibodies and reagents used for flow cytometry analysis included the anti‐CDH1 antibodies (rat IgG1 APC isotype control; Table S1), hMSC‐negative cocktail (isotype: PE hMSC isotype control negative cocktail), anti‐CD44 (isotype: PE mouse IgG2b isotype control) and hMSC‐positive cocktail (CD73‐APC, CD90‐FITC, CD105‐PerCP, isotype: hMSC‐positive isotype control cocktail; BD, cat# 562245).

### 2.5 EXMC subculture

The sorted EXMCs^LUM+^ and EXMCs^BST2+^ were cultured in medium consisting of DMEM/F12, 0.3% BSA (Sigma‐Aldrich, A3059, St. Louis, MO, USA), 0.2% FBS, 1% ITS‐X (100x, Gibco, 51,500,056), 1.5 mg/mL L‐ascorbic acid (Sigma‐Aldrich, A8960‐5G), 0.5 mM A83‐01 (Selleck, s7692), 1 mM SB431542 (Cellagentec, 3814, San Diego, CA, USA), 50 ng/mL hEGF (Miltenyi Biotec, 130–097‐750, Bergisch Gladbach, Germany), 2 μM CHIR99021, 0.8 mM valproic acid (Sigma‐Aldrich, V0033000), 0.1 mM beta‐mercaptoethanol (Sigma‐Aldrich, M3148‐25ML) and 5 mM Y‐27632^14^ at 37°C and 5% CO_2_ with saturated humidity. After reaching 80%–90% confluence, the cells were disassociated into single cells using the TrypLE treatment for 5 min at 37°C. The cells were routinely passaged and re‐plated on the Petri dishes (Corning, 430,166) precoated with 1% human collagen (Sigma‐Aldrich, C7521‐5MG) at a 1:3 split ratio every 3–4 days. The medium was changed every 2 days. All cell images were documented using DMIL LED Leica (LAS v4.9).

### 2.6 Differentiation of MSCs from EXMCs

After three passages, 10,000 EXMCs^LUM+^ were plated on 1% human collagen‐coated Petri dishes and cultured in serum‐free MSC medium (Yokon, NC0103, NC0103.S, Beijing, China) for MSC induction. The EXMC‐derived MSCs (E‐MSCs) were produced after three passages in MSC serum‐free medium. The E‐MSCs and UC‐MSCs as the control were cultured in MSC serum‐free medium. To passage E‐MSCs, 80%–90% confluent cells were disassociated into single cells using the TrypLE treatment for 3 min at 37°C and routinely passaged at a 1:4 split ratio every 3–4 days. The medium was changed every 2 days. The cells were cultured at 37°C and 5% CO_2_ with saturated humidity.

For the process of osteogenic differentiation, E‐MSCs were seeded in 1% gelatin‐coated 6‐well plates at a density of 2 × 10^4^ cells/cm^2^ with 2 mL of MSC serum‐free medium. When E‐MSCs reached 70% confluence, 2 mL of OriCell® MSC osteogenic differentiation medium (OriCell, HUXUC‐99021, Guangzhou, China) was added to the 6‐well plates. The medium was changed every 3 days. The cells were stained with alizarin red after 2–4 weeks of culture.

For chondrogenic differentiation, E‐MSCs (4 × 10^5^) were transferred to a 15‐mL centrifuge tube. After centrifugation (1000  r/min for 5 min at room temperature), the cells were resuspended in 0.5 mL of OriCell® chondrogenic differentiation complete medium (OriCell, HUXUC‐99041). The cells were then washed twice with the chondrogenic differentiation complete medium and centrifuged (1000 r/min for 5 min at room temperature) until they settled at the bottom of the centrifuge tube. The centrifuge tube cap was loosened to facilitate gas exchange. The cells were then cultured in suspension in the centrifuge tube. The medium was changed every 2–3 days. Alcian blue staining was performed when a cartilage sphere with a diameter of 1.5–2 mm formed in the tube.

For the process of adipogenesis differentiation, E‐MSCs were seeded in 1% gelatin‐coated 6‐well plates at a density of 2 × 10^4^ cells/cm^2^ with 2 mL of MSC serum‐free medium. When E‐MSCs reached 100% confluence, 2 mL of OriCell® MSC adipogenesis differentiation medium A (OriCell, HUXUC‐99021, Guangzhou, China) was added to the 6‐well plates. After 3 days, the medium was replaced by OriCell® MSC adipogenesis differentiation medium B. The induction and maintenance process were repeated until the appearance of appropriate lipid droplets was observed. The cells were stained with oil red O after 2–4 weeks of culture.

### 2.7 Immunofluorescence

To prepare frozen sections, EBs developed on different dates were collected into a 15‐mL centrifuge tube. The supernatant was removed after EBs were allowed to naturally settle for 5 min. The EBs were washed three times in PBS (5 min/wash), treated with precooled 4% paraformaldehyde for 1 h at 4°C, washed three times in PBS and resuspended in 20% sucrose solution at room temperature. After naturally settling at the bottom of the tube, the EBs were embedded using the optimal cutting temperature compound (OCT) and cooled on dry ice for 30 min. The frozen samples were cut into 10‐μm slices on the adhesion slide using a Leica freezing slicer. The slides were placed on a 37°C Leica heating plate for 30 min and stored at −20°C. For the immunofluorescence staining, the slides were washed three times in PBS to remove OCT and treated with 0.2% Triton X‐100 to permeabilize the membrane for 30 min at room temperature. After blocking with 3% BSA/PBS for 2 h, the slides were incubated with the primary antibody at 4°C overnight. The slides were then washed three times in 0.05% Tween‐20/PBS and incubated with the secondary antibody and 4′, 6‐Diamidino‐2‐phenylindole dihydrochloride (DAPI, 1:1000) for 2 h at room temperature in the dark. After washing three times in PBS, the slides were sealed with 50% glycerol. The images were obtained using Nikon AX/AX R Confocal Microscope (Nikon, Nikon AX, Tokyo, Japan). Primary antibodies used in the study are listed in Table S1.

### 2.8 Quantitativereal‐timePCR

Total RNA was isolated using the total RNA kit (Tiangen, DP419, Beijing, China) and cDNA synthesis was performed from total RNA using the hifair III 1st strand cDNA synthesis superMix for Qpcr (Yeasen, 1114ES60, Shanghai, China). Real‐time PCR was performed using Hieff UNICON Universal blue qPCR SYBR Maxter mix (Yeasen, 11184ES08) on the Step One Plus Real‐Time PCR System (Yeasen). All analyses were done in triplicate. Gene expression was normalised to β‐actin. Error bars represent the standard deviation (SD) of the mean of triplicate reactions. Primer sequences are included in the Table S2.

### 2.9 RNA‐Seq analysis

The 2 × 150 bp paired‐end libraries were sequenced using Illumina NovaSeq 6000. Library construction and sequencing were performed by Annoroad Gene Technology (http://www.annoroad.com/). The HISAT2 software (v2.2.1) was used to align paired‐end clean reads against the human reference genome (hg38). The StringTie (v2.1.1) was used for transcript assembly, General transfer format (GTF) document mergence and transcript abundance estimation. Next, the read count for each transcript was obtained from the coverage values using the prepDE.py Python script. The DESeq2 was used to analyse the differentially expressed genes (DEGs) (v1.30.1). Genes with a *p*‐value <0.05 and log2 (FoldChange) >1 were regarded as DEGs. R package ‘pheatmap’ (v1.0.12) was used for heat mapping of selected genes.

### 2.10 The scRNA‐Seq analysis

The D7 EBs were collected using natural sedimentation and washed three times in PBS. The cells were released from EBs using the TrypLE treatment for 20 min in a 37°C water bath and filtered using a 20‐μm filter. The filtered cells were resuspended in 0.1%FBS/PBS and treated with Trypan Blue (#1450013, Bio‐Rad) to assess cell viability. Cells with viability >95% were loaded into 10x Genomics Chromium (10x Genomics, Pleasanton, CA, USA). Library sequencing was performed by Annoroad Gene Technology (http://www.annoroad.com/) on an Illumina NovaSeq 6000 with 150‐bp paired‐end sequencing.

The RNA‐Seq analysis was carried out using the R software (version 4.2.0). The samples were loaded using Seurat software. NormalizeData and ScaleData functions were utilised to normalise and scale the data. FindIntegrationAnchors and IntegrateData functions were used to integrate the data. The data dimension was reduced by principal component analysis and cell types were defined based on specific gene expression. The normalised counts were then used to identify markers with the FindAllMarkers function. The plots were generated using R. DEGs between different cell types were determined using the FindMarkers function in Seurat, test.use with DESeq2 (1.32.0).^30^ The *p*‐value of <0.05 and min.pct = 0.1 were used to define significant differences in gene expression. Gene ontology (GO) and Kyoto Encyclopaedia of Genes and Genomes (KEGG) pathway analyses of DEGs were conducted using the Kobas website (http://bioinfo.org/kobas/genelist/). GO terms with a *p*‐value of <0.05 were defined as significantly enriched. Monocle (v2.18.0) was used to perform trajectory analysis and generate a heatmap for the cell type under consideration.

### 2.11 Label‐free semi‐quantitative proteomics analysis

The culture medium supernatants from third‐passage (P3) EXMCs^LUM+^ and UC‐MSCs were collected using trypsin digestion by sequential reduction of disulfide bonds with dithiothreitol and alkylation with methyl methanethiosulfonate. Then, the peptides were extracted and prepared for Liquid chromatography mass spectrometry (LC–MS/MS) (Thermo Fisher Scientific, Ultimate3000, Orbitrap Fusion™Lumos™Tribrid™ Mass Spectrometer, Waltham, MA, USA). The raw MS files were analysed and searched against the target protein database based on the sample species using Maxquant (1.6.2.10). The parameters were set as follows: the protein modifications were carbamidomethylation (C) (fixed), oxidation (M) (variable) and acetyl (protein N‐term) (variable); the enzyme specificity was set to trypsin; the maximum missed cleavages were set to 2; the precursor ion mass tolerance was set to 20 ppm and the MS/MS tolerance was set to 20 ppm. Only high‐confidence identified peptides were chosen for downstream protein identification analysis. R package ‘pheatmap’ (v1.0.12) was used for heat mapping of the selected proteins. Omicsbean (http://www.omicsbean.cn) was used to annotate UniProt based on the identified protein ID. The GO database annotation information for the corresponding protein was obtained from the UniProt database by mapping. GO term and protein–protein interaction (PPI) network analyses used Omicsbean.

### 2.12 Statistical analysis

**FIGURE 1:**
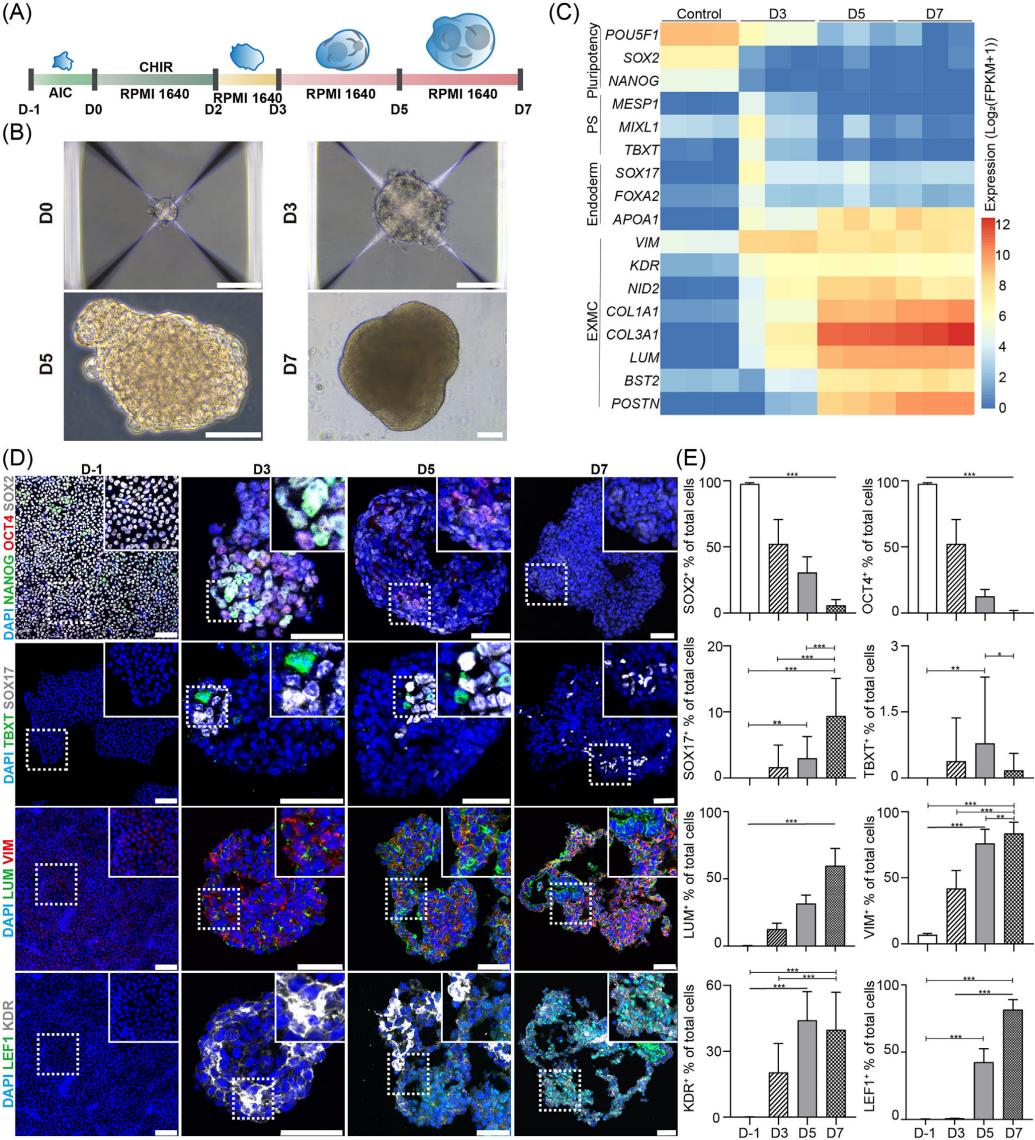
Differentiation of extraembryonic mesoderm cells (EXMCs) from human primed embryonic stem cells (ESCs). (A) Schematic diagram of the EXMC differentiation protocol. (B) Representative morphologies of hpESC‐derived embryoid bodies (EBs) on the indicated day from day 0 (D0) to day 7 (D7). Scale bar: 100 μm. (C) Heatmap of lineage‐specific gene expression in EBs on different differentiation days. Gene expression levels were normalised. (D) Representative immunostaining images of lineage‐specific markers in EBs on different days, including NANOG, OCT4 and SOX2 for pluripotency; TBXT for mesoderm; SOX17 for endoderm; LUM, VIM, LEF1 and KDR for EXMC. Scale bars: D‐1, 100 μm; others, 50 μm. (E) Immunostaining images of lineage marker quantification. One‐way ANOVA for multiple comparisons. *n* = 3 in each treatment. Data are represented as mean ± sd. *p < 0.05, **p < 0.01 and ***p < 0.001.

**FIGURE 2:**
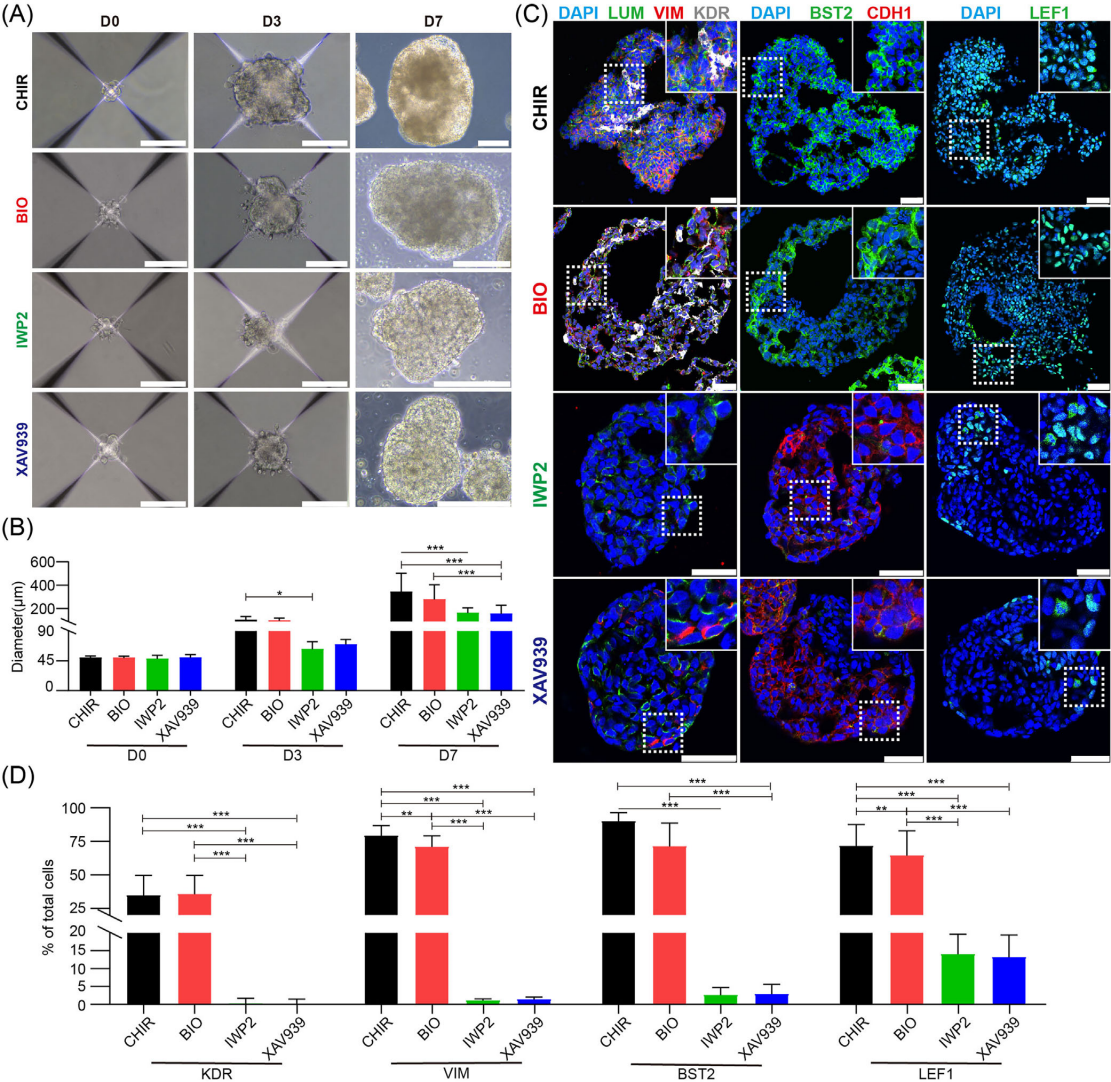
Wnt signalling promotes EXMC generation. (A) Representative phase‐contrast images of embryoid bodies (EBs) treated with CHIR99021 (CHIR), BIO, IWP2 and XAV939 at different time points (D0, D3 and D7). Scale bar: D0, D3: 100 μm; D7: 200 μm. (B) Quantification of diameters of EBs treated with CHIR, BIO, IWP2 and XAV939 at different time points. *n* = 3 in each group. (C) Representative immunostaining images of lineage‐specific markers in D7 EBs treated with CHIR, BIO, IWP2 and XAV939. Scale bars: 50 μm. (D) Quantification of lineage marker (KDR, VIM, BST2 and LEF1)‐positive cells in D7 EBs. B, D, Data are represented as mean ± sd. *n* = 3 in each group. Two‐way ANOVA for multiple comparisons were performed. *p < 0.05, **p < 0.01 and ***p < 0.001.

Statistical testing and data processing were performed in R (v4.2.0). Wilcoxon test was used to calculate the statistical significance of gene expression in scRNA‐Seq. Information on each statistical test and multi‐test correction can be found in the Results section and Figure Legends. The samples were tested three times at each time point for the statistical evaluation of the RNA‐Seq experiments. All experiments were repeated at least three times for h1 and h2 human ESCs, EXMCs and E‐MSCs. One‐way ANOVA (Figures 1E and S1D,G) was used to determine the statistical differences in the differentiation efficiency of EXMCs. Two‐way ANOVA (Figures 2B,D and S2C) was used for multiple comparisons to analyse the differences between groups. *p < 0.05 indicated that the difference was statistically significant, **p < 0.01 showed that the difference was highly statistically significant and ***p < 0.001 demonstrated that the difference was extremely statistically significant. All quantification data were represented as the means ± SD.

## 3 RESULTS

### 3.1 EXMC differentiation from hpESCs

According to the previous studies, activation of the Wnt pathway is known to induce the differentiation of EPI into the PS stage, sequentially initiating the formation of the three germ layers. Although the Wnt pathway is involved in the initiation of various lineage differentiations, the key role of Wnt signalling during hpESC differentiation into EXMCs remains unknown. By exploring the single‐cell transcriptome data for human embryos from day 11 to day 14,^6^ it was discovered that the Wnt signalling genes^31^ were highly expressed in EXMCs (Figure S1A). Although the *CTNNB1* gene that encodes beta‐catenin is universally expressed in all post‐implantation embryonic lineage cells, both *FZD2* that encodes frizzled‐2 receptor of the canonical signalling pathway and *LEF1* that is a downstream mediator of the Wnt/β‐catenin signalling pathway were specifically expressed in EXMCs (Figure S1B). This observation showed that the Wnt signalling pathway may be required for the generation of EXMCs in vitro.

A highly selective GSK‐3 inhibitor for canonical Wnt signalling activation CHIR99021 (CHIR) was utilised to investigate whether the Wnt activator promoted EXMC differentiation in the EB culture medium. EBs were treated with CHIR for 24, 36, 48 and 72 h, collected on D3, D5 and D7 and immunologically stained using well‐known EXMC markers KDR and VIM.^14^ The results showed that the KDR‐positive EXMC percentage increased from 26.14 ± 6.62–31.60% ± 6.45% (CHIR‐24 h to CHIR‐36 h) to 36.98% ± 7.77% (CHIR‐48 h) on D7 (Figure S1C,D) as the duration of the CHIR treatment increased. Similarly, the VIM‐positive EXMC percentage increased from 51.25 ± 6.80–69.10% ± 3.30% (CHIR‐24 h to CHIR‐36 h) to 80.31% ± 4.27% (CHIR‐48 h) on D7 (Figure S1D). Interestingly, prolonged CHIR treatment (CHIR‐72 h) caused a decrease of VIM‐ (65.60% ± 8.90%) and KDR‐positive cells (20.50% ± 3.65%) on D7, compared to the CHIR‐48 h treatment (Figure S1C,D). These data imply that EXMC production is dependent on the duration of CHIR treatment. In the gene expression profile, the expression level of pluripotent genes (*NANOG*, *OCT4*) decreased significantly as the duration of the CHIR99021 treatment increased. At the same time, the expression of EXMC‐specific genes (*LUM*, *BST2*) increased significantly, suggesting that the Wnt signalling pathway plays a key role in promoting the differentiation of hpESCs into EXMCs (Figure S1E).

Because the 48‐h CHIR treatment of EBs provided the optimal conditions to promote EXMC differentiation, the following differentiation protocol to generate EXMCs was adopted (Figure 1A). After seeding in the AggreWell plate on D0, hpESCs aggregated on the bottom of the plate and formed spherical shapes. The cell aggregates cultured in the EXMC medium were then gradually developed and converted to EBs with asymmetric structures between D3 and D7 (Figure 1B). The RNA‐Seq analyses showed that hpESC‐derived EBs highly expressed the EXMC genes (such as *VIM*, *KDR* and *COL3A1*) but clearly impeded the expression of pluripotent genes in D5 and D7 EBs (Figure 1C). Consistent with the RNA‐Seq data, the immunostaining results showed that the numbers of LUM‐, VIM‐, KDR‐ and LEF1‐positive cells were significantly increased, accompanied by a significant decrease in OCT4‐ and SOX2‐positive cells between D3 and D7^32^ (Figure 1D,E). KDR‐positive cells were significantly lower than VIM‐positive cells (Figure 1D,E), suggesting that KDR‐positive cells may be one subtype of EXMC. The numbers of cells positive for the endoderm marker SOX17 also increased in D7 EBs compared to the early stages of EBs. However, their numbers were close to or less than 10% of total cells. The numbers of cells positive for the mesoderm marker TBXT failed to exhibit a consistent increase in D3, D5 and D7 EBs. After CHIR treatment, we detected the activation of beta Catenin (CTNNB1) and the Wnt downstream genes (*CTNNB1, FZD2* and *LEF1*) on D1 and D3 (Figure S1F,G). Together, these results indicated that the differentiation protocol efficiently induced hpESCs to differentiate into EXMCs via EB formation.

### 3.2 Wnt signaling pathway promotes EXMC generation

Three chemical compounds including BIO (a specific GSK‐3 inhibitor), XAV939 (a potent inhibitor for the canonical Wnt signalling pathway) and IWP2 (a universal Wnt signalling pathway inhibitor) were applied in the differentiation medium to further verify whether the Wnt pathway activation is required for EXMC generation. The results showed that the IWP2 and XAV939 treatment hampered the growth and development of EBs compared to the CHIR and BIO treatment, suggesting that inhibition of the Wnt signalling pathway visibly affected EB formation (Figure 2A). The EB diameters in the XAV939 (154.30 ± 36.63 μm) and IWP2 (160.90 ± 22.88 μm) groups were significantly smaller than those in the CHIR and BIO group (342.0 ± 79.60 μm and 277.63 ± 62.64 μm) on D7 (Figure 2B). The immunostaining results showed that most of the cells preserved the pluripotent markers in IWP2‐ and XAV939‐treated D3 EBs (Figure S2A). The OCT4‐ and SOX2‐positive cells were almost undetected in the CHIR‐ and BIO‐treated D7 EBs, but 28.47 ± 4.84% and 23.99 ± 4.42% of cells were present in IWP2‐treated EBs and 23.33 ± 4.37% and 18.96 ± 5.72% of cells were present in the XAV939‐treated EBs, respectively (Figure S2B, C). As the EXMC markers, KDR‐positive cells were almost absent in both the IWP2 and XAV939 groups (Figure 2C,D). The VIM‐ and BST2‐positive cells were present at a rate of <3% in both the IWP2 and XAV939 groups (Figure 2C,D). In the CHIR and BIO groups, 69.56% ± 8.34% and 64.33% ± 9.28% of cells were positive of LEF1 respectively, in agreement with the fact that *LEF1* was involved in the Wnt pathway and is specifically expressed in EXMCs (Figures S1B and S3C). However, when the Wnt pathway was blocked, the proportions of LEF1‐positive cells decreased to 14.02% ± 2.80% in the IWP2 group and 13.22% ± 3.10% in the XAV939 group (Figure 2C,D). Moreover, TBXT‐positive cells appeared in both IWP2‐ (3.65 ± 1.29%) and XAV939‐treated (0.80% ± 0.67%) D7 EBs, but were undetected in both CHIR‐ and BIO‐treated EBs (Figure S2B,C). In addition, SOX17‐positive cells were only detected in the XAV939 group (2.65 ± 0.83%), but were absent in the IWP2 group, suggesting that hpESCs maintained their capability to differentiate toward endoderm lineage cells when the canonical Wnt signalling pathway was blocked (Figure S2B,C). Together, these results showed that the Wnt pathway inhibition significantly impeded the hpESC differentiation toward EXMCs in EBs.

### 3.3 Transcriptome profiling of EXMCs

**FIGURE 3:**
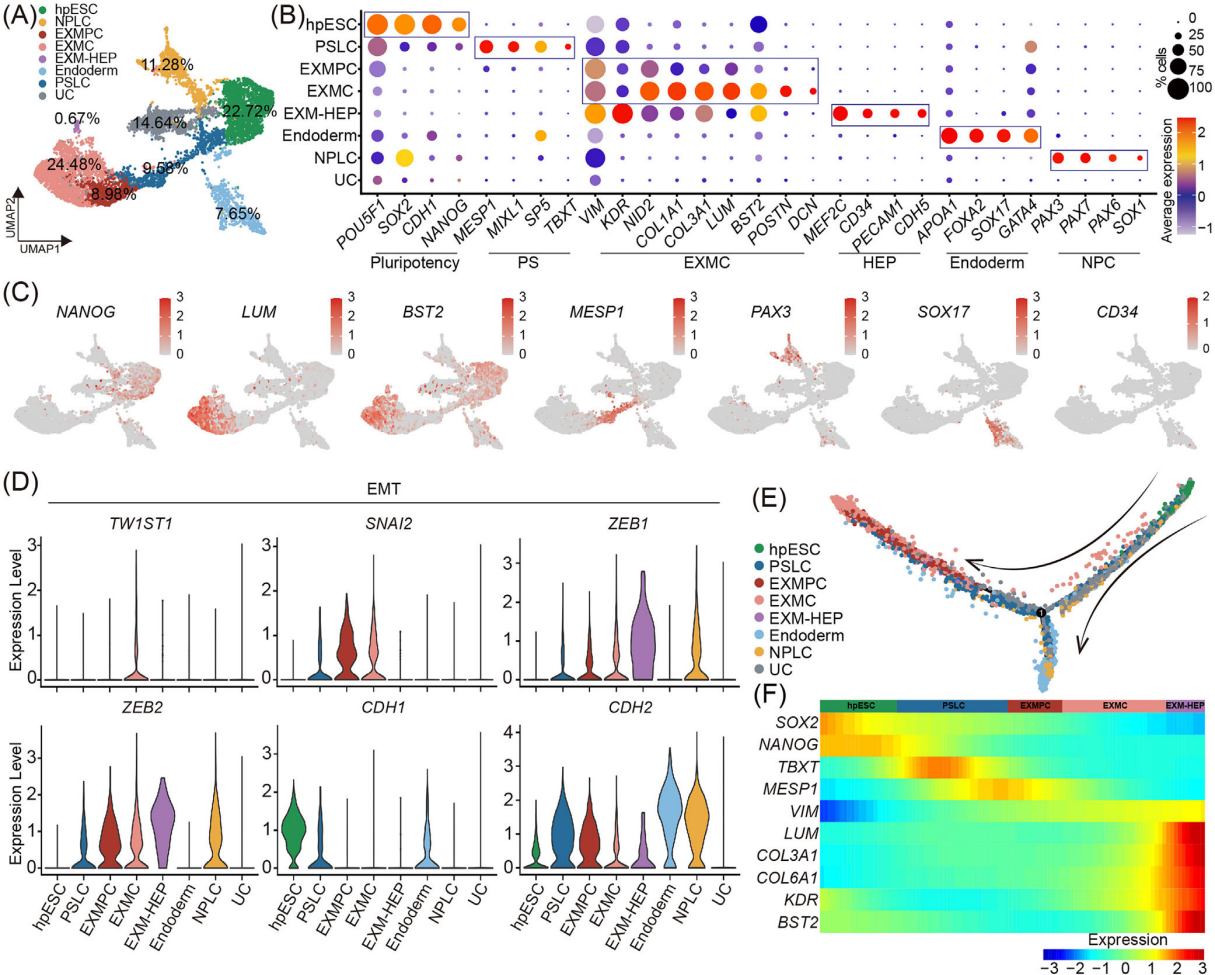
Transcriptomic profile for extraembryonic mesoderm cells (EXMCs). (A) Uniform manifold approximation and projection (UMAP) plot of single‐cell transcriptomic data for D7 embryoid bodies (EBs). (B) Dot plots for lineage‐specific genes expressed in different cell types. Dot sizes indicate the proportion of cells that express a given gene in all clusters. Gene expression level is shaded by colour from low (blue) to high (red). (C) Expression of lineage‐specific genes in different cell clusters. (D) Violin plot for EMT‐related gene expression in different cell clusters. (E) Pseudotime analysis of different cell types in D7 EBs. (F) Heatmap showing the dynamics of key markers during hpESC progression via primitive streak‐like cells (PSLCs) to EXMCs based on pseudotime analysis.

The scRNA‐Seq analysis was performed on the collected D7 EBs to illustrate the transcriptomic profile of EXMCs. After strict filtering, a total of 8353 cells from D7 EBs were annotated into eight distinct cell types using canonical lineage‐specific markers (Figure S3A). The eight cell types included hpESCs, primitive streak‐like cells (PSLCs), EXMC precursor cells (EXMPCs), EXMCs, EXMC‐hemogenic endothelial progenitors (EXM‐HEPs), endoderm cells, neural progenitor‐like cells (NPLCs) and undefined cells (UCs). EXMCs (24.48% of EXMCs, 8.98% of EXMPCs and 0.067% of EXM‐HEPs) constituted one‐third of the cell population in D7 EBs (Figure 3A). There were still 22.72% of undifferentiated hpESCs and 9.58% of PSLCs in the D7 EBs (Figure 3A), suggesting that the differentiation culture conditions may need further optimization to increase EXMC production. Lineage‐specific genes for each cell type are itemised in Figure 3B. EXMC‐specific *LUM* and *BST2* genes were highly expressed in the EXMC and EXMPC clusters, whereas the pluripotent *NANOG* gene was expressed in the hpESC cluster. Interestingly, a few cells co‐expressed *KDR* and *CD34*, which was defined as a subtype of EXMCs for EXM‐HEPs (Figure 3B). This may explain that the number of KDR‐positive cells is less than VIM‐positive cells (Figures 1D,E and 2C, D). The expression of lineage‐specific genes, such as *MESP1* for PSLCs, *PAX3* for NPCs and *SOX17* for endoderm cells was also verified in the subclusters (Figure 3C). KEGG pathway analysis showed that cells in the EXMC cluster expressed focal adhesion function‐related genes, including *COL4A2*, *COL4A1*, *LAMC1* and *FLNC*, indicating that EXMCs gain the extracellular secretion competency (Figure S3A). Amnion cell marker *ISL1* and trophoblast cell marker *KRT7* ^11^ were not detected in the D7 EBs, suggesting that culture conditions for EXMC differentiation did not induce the development of extraembryonic ectodermal cells and trophoblasts (Figure S3B).

The expression of EMT‐related genes within different cell types was analysed because EXMCs initially underwent the epithelial‐to‐mesenchymal transition (EMT) during embryo development. The results showed that EMT‐related genes^33^ *TW1ST1* and *SNAI2* were highly expressed in EXMPC and EXMC cell clusters, whereas the EMT genes *ZEB1*, *ZEB2* and *CDH2* were highly expressed in all initially differentiated cell clusters except hpESCs and the endoderm (Figure 3D). Pseudotime trajectory analysis was carried out to further understand the developmental and differentiation trajectories of cells in the D7 EBs. The analysis results revealed that the single‐cell data trajectory from hpESCs to EXMCs had an inflexion point, where the EXMCs exhibited a different developmental direction against the endoderm and NPLCs (Figure 3E). The PSLCs were situated in both EXMC and NPLC directions, suggesting that PSLCs as an intermediate state may bridge hpESC differentiation into EXMCs. A clear differentiation progression from hpESCs through PSLCs to EXMCs was evident when hpESCs, PSLCs, EXPMCs, EXMCs and EXM‐HEPs were lined up on the heatmap (Figure 3F). Therefore, increasing the proportion of PSLCs may help to increase the EXMC differentiation rate.

### 3.4 Comparison of EXMCs with embryonic and naïve‐dreived EXMCs

**FIGURE 4:**
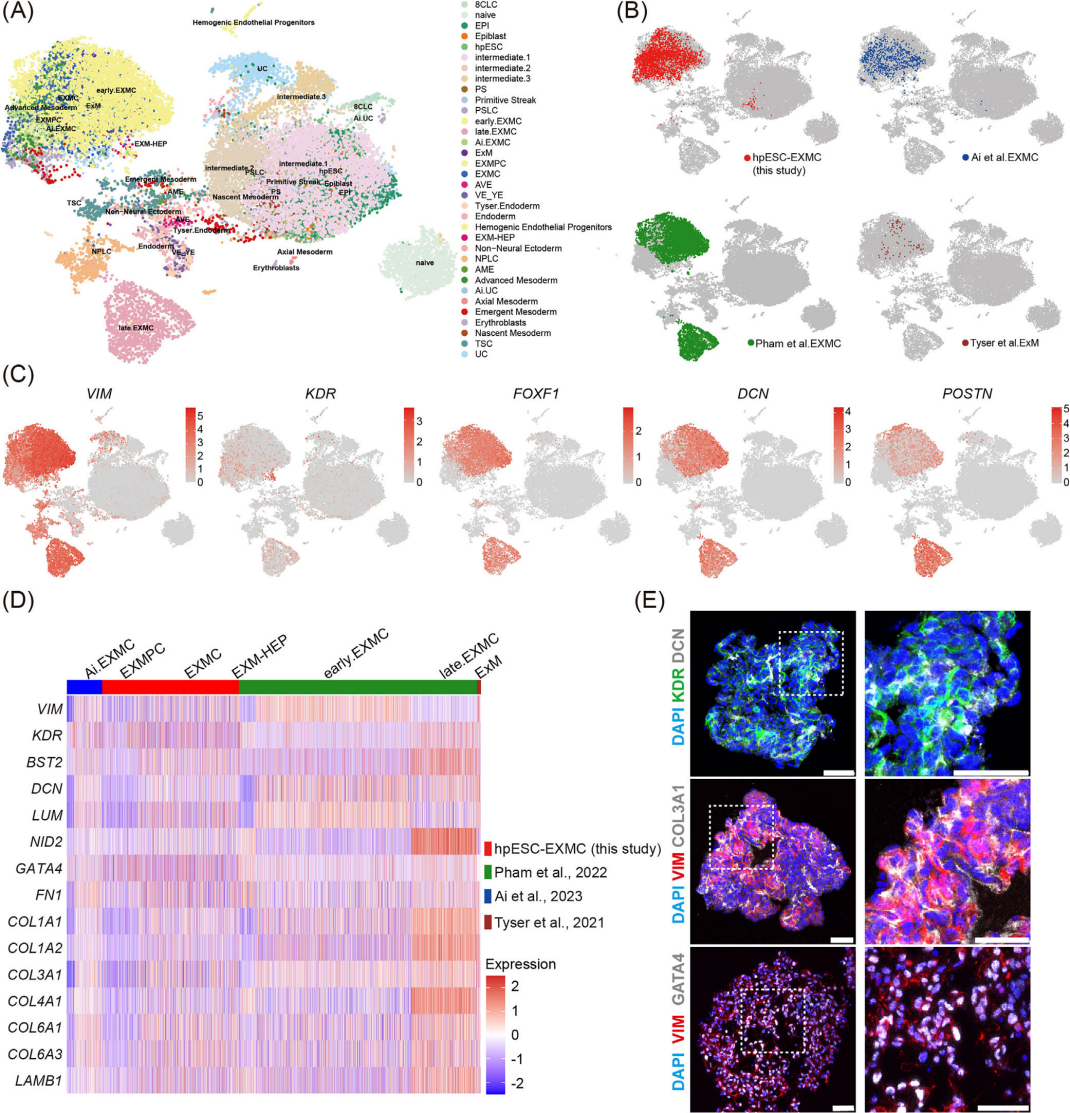
Comparative analysis of the reported extraembryonic mesoderm cells (EXMCs). Integrative analysis of single‐cell RNA‐Seq data from EXMCs and three other reported databases, including naïve hESC‐derived EXMC data (Pham_EXMC),^14^ post‐implantation embryo‐derived EXMC data (E10‐E14, Ai_EXMC),^6^ and gastrula stage ExM data (E16‐E19, Tyser_ExM).^32^ (A) Uniform manifold approximation and projection (UMAP) showing scRNA‐Seq integrated data from four different databases annotated into 34 cell clusters based on the canonical lineage markers and differentially expressed genes (DEGs). (B) Individual EXMC population visualisation on integrated UMAP. (C) EXMC‐specific gene expression in all cell clusters. (D) Heatmap of genes in different clusters based on integrated scRNA‐Seq data. (E) Representative immunostaining images of markers in D7 embryoid bodies (EBs). Scale bar: 50 μm.

Several sets of scRNA‐Seq data for human EXMCs have been recently reported, including human naïve PSC‐derived EXMCs (Pham et al. 2022, Pham_EXMC),^14^ post‐implantation embryo‐derived EXMCs (Ai et al., 2023, Ai_EXMC),^6^ and human gastrulating embryo ExM (Tyser et al. 2021, Tyser_ExM).^34^ To further assess the EXMCs, the scRNA‐Seq data for hpESC‐derived EXMCs (hpESC‐EXMCs) were used for a comprehensive comparison with the reported scRNA‐Seq data. The dataset integrated eight cell types from EXMCs (hpESC, NPLC, PSLC, EXMPC, EXMC, EXM‐HEP, endoderm and UC), seven cell types from Ai_EXMC (EPI, VE_YE, AVE, AME, PS, Ai_EXMC and Ai_UC), 11 cell types from Tyser_ExM (Advanced Mesoderm, Axial Mesoderm, Emergent Mesoderm, Tyser_Endoderm, EPI, Erythroblast, ExM, HEPs, Nascent Mesoderm, Non‐Neural Ectoderm and Primitive Streak) and eight cell types from Pham_EXMC (8CLC, naïve, intermediate.1, intermediate.2, intermediate.3, TSC, early.EXMC and late.EXMC) (Figure 4A). As shown on a uniform manifold approximation and projection (UMAP) (Figrue 4B), hpESC‐EXMCs showed a high level of correspondence with Ai_EXMCs, Tyser_ExM and early phase of Pham_EXMCs. However, hpESC‐EXMCs, Ai_EXMCs and Tyser_ExM demonstrated almost no overlap with the late phase of Pham_EXMCs (Figure 4B). Thus, it is worthwhile to monitor the cell identity of late phase Pham_EXMCs. The UMAP showed the specific gene markers for different cell clusters. *VIM* was highly expressed in EXMC and NPLC clusters (Figure 4C). LUM, FOXF1 and DCN were highly expressed in EXMCs, while the *POSTN* and *NID2* expression values were higher in the late phase than in the early phase of Pham_EXMCs (Figure 4C, S4A). *KDR* was mainly expressed in the EXM‐HEP cluster (Figure 4C). ECM‐specific genes (*FN1*, *COL1A1*, *COL1A2*, *COL3A1*, *COL4A1*, *COL6A1*, *COL6A3* and *LAMB1*) were detected and expressed in hpESC‐EXMCs, similar to other reported EXMCs (Figure 4D). Gene expression results from scRNA‐Seq data were further verified using immunostaining assays. COL3A1, VIM, DCN and GATA4 were spotted on the differentiated cells from D7 EBs (Figure 4E). In summary, transcriptomic results revealed that the hpESC‐EXMCs showed extensive similarity to the reported EXMCs, especially with the early phase of Pham_EXMCs.

### 3.5 EXMC purification and expansion

**FIGURE 5:**
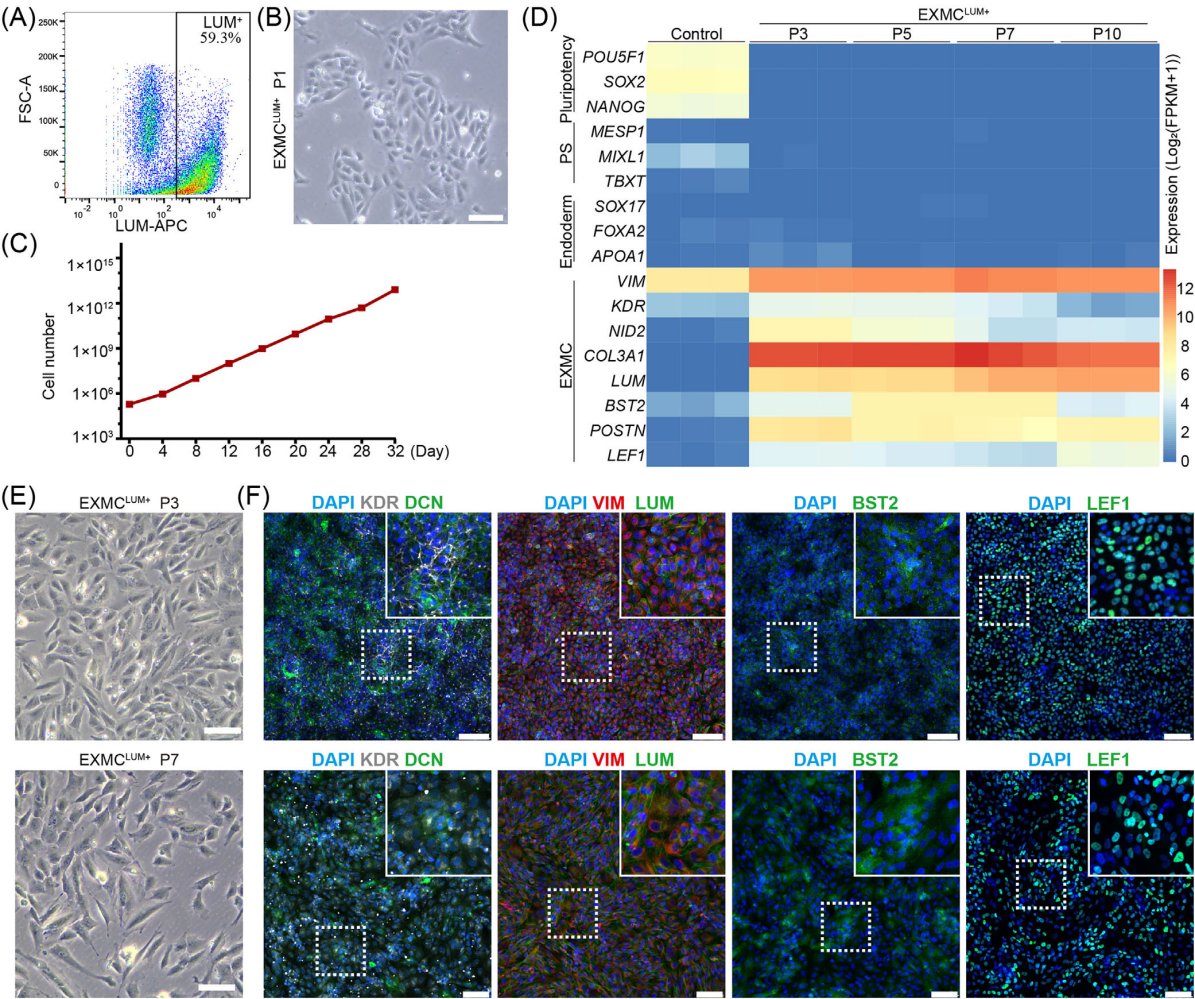
EXMC sorting and expansion. (A) Flow cytometry sorting of extraembryonic mesoderm cells (EXMCs^LUM+^) from D7 embryoid bodies (EBs). (B) Representative morphologies of EXMCs^LUM+^ subcultured at passage one (P1). Scale bar: 100 μm. (C) Cell proliferation curves for EXMCs^LUM+^. (D) Heatmap of lineage‐specific gene expressions in control (hpESC, D‐1) and EXMCs^LUM+^ at P3, P5, P7 and P10. Gene expression levels were normalised. (E) Representative morphologies of EXMCs^LUM+^ at P3 and P7. Scale bar: 100 μm. (F) Representative immunostaining images of markers expressed in EXMCs^LUM+^ at P3 and P7. Scale bars: 100 μm.

Previous studies have shown that some EXMCs were observed during long‐term TSC passage,^14^ suggesting that the TSC medium can support EXMC expansion. According to the scRNA‐Seq data, *LUM*‐ and *BST2‐*positive cells were distributed in the EXMC cluster (Figure 3C). Flow cytometry results showed that the percentage of LUM‐positive cells was increased from 13.1% in D3 EBs to 34.5% in D5 EBs. The percentage of BST2‐positive cells was increased from 15.6% in D3 EBs to 57.2% in D5 EBs (Figure S4B). To purify EXMCs, LUM‐positive EXMCs (EXMCs^LUM+^; 59.3%) from D7 EBs were first sorted using fluorescence‐activated cell sorting (FACS) (Figure 5A) and the enriched EXMCs^LUM+^ were seeded in the EXMC medium (TSC medium)^14^ as the first generation (P1) (Figure 5B). Purified EXMCs were cultured in vitro for multiple passages (Figure 5C). RNA‐Seq analysis revealed that EXMCs^LUM+^ highly expressed the EXMC‐specific genes (*VIM*, *NID2*, *COL3A1*, *LUM*, *BST2*, *POSTN* and *LEF1*) during long‐term expansion (Figure 5D). EXMCs^LUM+^ exhibited typical front‐back polarity morphology during long‐term passage (Figure 5E). The immunostaining showed that EXMCs^LUM+^ maintained the EXMC characteristics (Figure 5F). BST2‐positive cells were also sorted, showing that 94.6% of cells were positive for BST2 (Figure S4C). These sorted BST2^+^ cells were continuously cultured in vitro and showed heterogeneous morphologies (Figure S4C).The immunostaining and FACS analyses showed that these cells only partially expressed EXMC markers KDR, DCN, COL3A1 and BST2 at P7 (Figure S4D). The BST2^+^/CDH1^−^ cells only accounted for 32% of the total cell population, suggesting that BST2 may not be a proper marker to purify EXMCs (Figure S4E). Purified EXMCs^LUM+^ maintained EXMC characteristics up to at least P10 (Figure S4F). Taken together, the study data showed that LUM was a suitable marker for sorting EXMCs and that EXMCs^LUM+^ could be expanded long‐term in vitro.

### 3.6 ECM protein production in EXMCs

**FIGURE 6:**
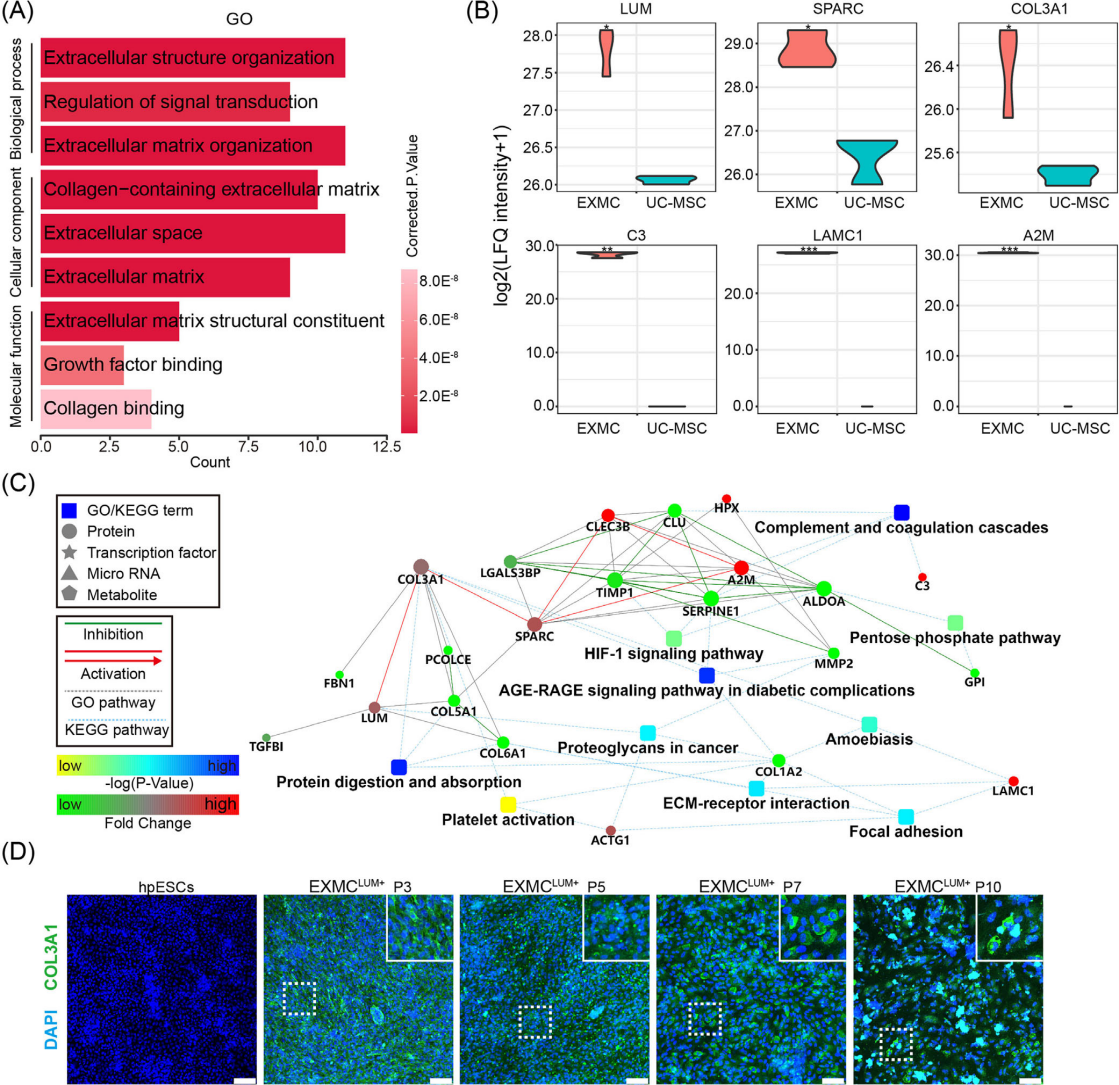
Expression and secretion of extracellular proteins in extraembryonic mesoderm cells (EXMCs). (A) Representative gene ontology (GO) terms and proteins highly enriched in EXMCs. (B) Violin plot of indicated extracellular protein expression level in medium supernatant of EXMCs^LUM+^ and umbilical cord MSCs (UC‐MSCs) at P3. *p < 0.05, **p < 0.01 and ***p < 0.001. (C) Protein–protein interaction (PPI) network of proteins highly enriched in EXMCs. (D) Representative immunostaining images of COL3A1 in EXMCs from the different passages. hpESCs were used as the control. Scale bars: 100 μm.

To explore the production of extracellular proteins, the supernatant from EXMCs^LUM+^ at P3 was collected and analysed using label‐free quantitative proteomics. The UC‐MSCs at P3 were used as the control since MSCs produced the ECM proteins.^35^ GO analysis showed the differences between EXMCs^LUM+^ and UC‐MSCs in biological processes, cellular components and molecular functions (Figure 6A). The biological processes demonstrated that EXMCs^LUM+^ were involved in extracellular structure organisation, regulation of signal transduction and ECM organisation. The cellular component revealed that EXMCs^LUM+^ were involved in collagen‐containing ECM, extracellular space and ECM. The molecular function results showed that EXMCs^LUM+^ took part in ECM structural constituents, growth factor binding and collagen binding (Figure 6A). GO pathway results showed that LUM, SPARC, COL3A1, C3, LAMC1 and A2M proteins were significantly upregulated in EXMCs (Figure 6B). A PPI network was also mapped, in which COL3A1 activated LUM followed by COL6A1 during ECM‐receptor interaction and focal adhesion (Figure 6C). EXMCs^LUM+^ between P3 and P10 were immunostained to verify the expression of COL3A1. The results showed that EXMCs^LUM+^ continuously expressed COL3A1 up to P10 (Figure 6D). Taken together, these outcomes demonstrated that EXMCs secreted a large amount of ECM proteins (collagen, laminin) and produced more COL3A1 than UC‐MSCs.

### 3.7 EXMC‐derived MSC characterisation

**FIGURE 7:**
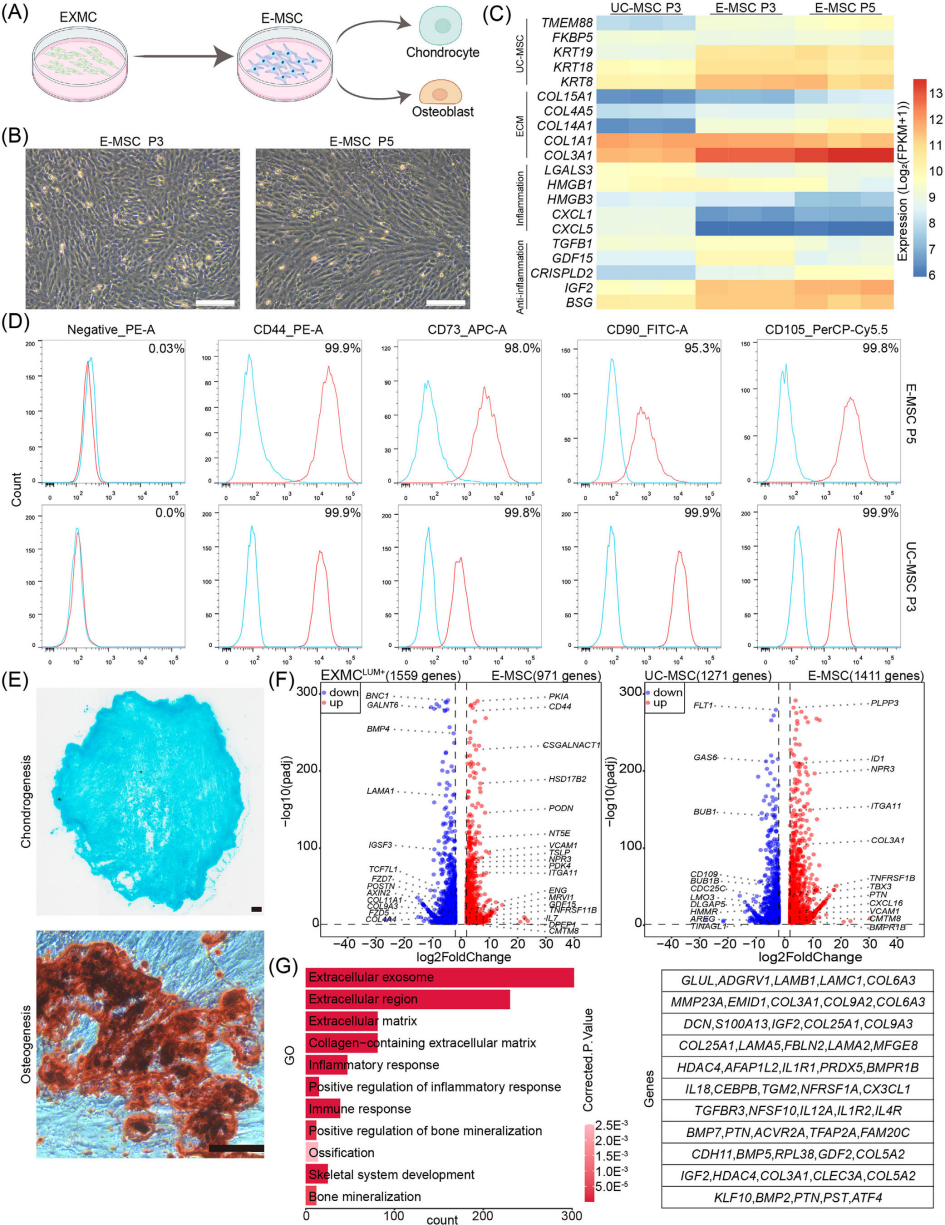
Characterisation analysis of EXMC‐derived E‐MSCs. (A) Schematic diagram of EXMC differentiation into E‐MSCs. (B) Representative morphologies of E‐MSCs at P3 and P5 in contrast‐phase field. Scale bars: 200 μm. (C) Expression heatmap of genes in P3 umbilical cord MSCs (UC‐MSCs) as the control, P3 E‐MSCs and P5 E‐MSCs. Gene expression levels were normalised. (D) Flow cytometry assays for MSC‐specific markers CD44, CD73, CD90 and CD105 in P5 E‐MSCs and P3 UC‐MSCs. (E) Representative images of chondrocytes (Alcian blue) and osteocytes (Alizarin red) differentiated from E‐MSCs. Scale bars: 200 μm. (F) Volcano plot of differentially expressed genes (DEGs) between extraembryonic mesoderm cells (EXMCs^LUM+^) and E‐MSCs (left panel), UC‐MSCs and E‐MSCs (right panel). (G) Representative gene ontology (GO) terms and genes highly enriched in E‐MSCs.

EXMC^LUM+^ differentiation was induced to verify the differentiation potential of EXMCs. The results showed that EXMCs differentiated into MSCs in the MSC medium^36^ (Figure 7A). The EXMC‐derived MSCs (E‐MSCs) were continuously cultured for five passages and exhibited spindle‐shaped morphology, which is typical for UC‐MSCs (Figure 7B). The alignment of RNA‐Seq data from UC‐MSCs (three independent lines) at P3, E‐MSCs in P3 and E‐MSCs in P5 showed that the MSC‐related genes (*KRT18* and *KRT8*), ECM‐related genes (*COL1A1* and *COL3A1*) and anti‐inflammation genes (*IGF2* and *BSG*) were highly expressed in both E‐MSCs and UC‐MSCs (Figure 7C). The FACS analysis showed that P5 E‐MSCs continued to express MSC‐specific markers CD44 (99.9%), CD73 (98%), CD90 (95.3%) and CD105 (99.8%), equivalent to UC‐MSCs (Figure 7D). The cell proliferation rates of UC‐MSCs and E‐MSCs were similar during passaging in vitro (Figure S4G). Interestingly, E‐MSCs could be induced to differentiate into chondrocytes and osteocytes, but not into adipocytes (Figure 7E; S4H). For osteogenic differentiation in vitro, GO analysis showed that compared to UC‐MSCs, the upregulated genes in E‐MSCs were related to cartilage development, bone mineralisation, chondrocyte differentiation and skeletal system development (Figure S4I), confirming stronger abilities of chondrogenesis and osteogenesis.

DEGs between E‐MSCs and EXMCs were compared to further characterise E‐MSCs. A total of 2530 DEGs (1559 downregulated and 971 upregulated genes in EXMCs) were observed. Compared to EXMCs, E‐MSCs upregulated MSC‐related (*CD44*, *NT5E*, *VCAM1* and *ENG*) and immune response and regulation (*TSLP*, *ITGA11*, *TNFRSF11B* and *IL7*) genes (Figure 7F). DEGs between E‐MSCs and UC‐MSCs were also compared. As a result, 2682 DEGs (1271 downregulated genes and 1411 upregulated genes in E‐MSCs) were noted. Interestingly, upregulated genes in E‐MSCs were enriched with immune response and regulation (*ITGA11*, *TNFRSF11B* and *CXCL16*) and bone tissue development (*TBX3*, *PTN*, *COL3A1* and *BMPR1B*) genes (Figure 7F). GO analysis showed that E‐MSCs were involved in ECM secretion, immune response and regulation, bone development and mineralisation (Figure 7G). In summary, E‐MSCs showed substantial similarities to UC‐MSCs in terms of gene expression, but demonstrated stronger abilities in ECM secretion, immune response and regulation and chondrocyte and osteocyte differentiation compared to UC‐MSCs.

## 4 DISCUSSION

EXMCs emerge in human embryos at Carnegie stage 5 during the initial stages of primate embryonic development, subsequently adopt other lineages to provide a niche for their development and eventually develop into the primitive umbilical cord in the stalk region.^19^, ^37^, ^38^, ^39^, ^40^ EXMCs are the earliest source of umbilical cord and MSCs isolated from the umbilical cord have important application values in regenerative medicine.^41^, ^42^, ^43^, ^44^ Therefore, it is crucial to establish an in vitro EXMC differentiation system, especially for exploring EXMC secretion and differentiation and EXMC‐derived MSC functions.

Recent studies on the establishment of EXMC models in vitro have been carried out using naïve PSCs or naïve PSC‐induced primitive endoderm cells (n‐EXMCs).^14^, ^45^ Because naïve ESCs are prone to genomic instability and aberrant methylation over expansion,^46^ these drawbacks limit the potential applications of naïve‐derived EXMCs. In the present study, EXMCs were first generated from hpESCs to obtain purified EXMCs with long‐term expansion ability. RNA‐Seq analysis and existing data comparisons demonstrated that EXMCs exhibited a significant similarity in gene expression profiles to those of embryonic EXMCs and n‐EXMCs in vitro.^6^, ^14^, ^34^ Therefore, the present study described a novel way to induce EXMC differentiation using EB suspension culture and to subsequently obtain high purity EXMCs. The study findings also provided an important platform to study developmental mechanisms and origins of EXMCs and MSCs.

Previous studies have shown that the Bmp pathway^11^, ^12^, ^14^ was essential for EXMC differentiation in vitro by activating the Wnt pathway, which was also involved in promoting other cell lineage differentiation in human post‐implantation embryos, such as EPI, mesoderm, endoderm and trophoblast.^47^, ^48^, ^49^, ^50^ In an embryo‐like assembloid generated by naïve hESCs and extraembryonic cells, continuous activation of the Bmp and Wnt signals induced naïve human ESCs into EXMCs.^6^, ^51^ These studies showed that the Bmp and Wnt signals were important for EXMC specification and development. In the present study, scRNA‐Seq analysis showed that genes (*FZD2*, *LEF1*) in the Wnt pathway were highly expressed in embryonic EXMCs (Figure S1A,B). EXMC differentiation was significantly inhibited in the present study after addition of the Wnt inhibitors IWP2 and XAV939 for 48 h. The study results confirmed that the Wnt pathway played an important role in EXMC generation. By transiently activating the Wnt pathway,^52^ hpESCs underwent the PS stage to generate EXMCs through self‐organisation. The pseudotime trajectory analysis showed that PSLCs were in the transitory stage between hpESCs and EXMCs (Figure 3E,F). WNT signalling inhibition significantly impeded PSLC and EXMC production (Figures 2D and S2A). These data suggest that EXMC production from hESCs may be through PSLC. However, it still needs to figure out whether the emergence of EXMCs is independent on the TBXT‐positive cells.

Identifying specific biomarkers to purify cells is one of the important challenges in stem cell research. The present study scRNA‐Seq data showed that *LUM* was mainly expressed in EXMPCs and EXMCs, while *BST2* was expressed in hpESCs, EXMCs and EXM‐HEPs (Figure 3B). To test whether the two markers could be used to purify EXMCs, EXMCs were isolated by sorting LUM‐ and BST2‐positive cells and cultured in the EXMC medium using previously established systems.^14^ The EXMCs^LUM+^ maintained EXMC characteristics during long‐term culture. In contrast, the BST2^+^ cells were separated into two different cell groups at P7. These results showed that LUM alone was enough to purify EXMCs as a marker.

Label‐free quantitative proteomics analysis was used to identify the function of EXMCs in vitro. The results showed that EXMCs produced many ECM proteins similar to UC‐MSCs.^35^, ^53^ In addition, EXMCs generated more COL3A1 than UC‐MSCs in the ECM‐receptor interaction and focal adhesion pathway. The secretory characteristics were consistent with the functions of EXMCs, including migration and tissue remodelling. Second, EXMCs gave rise to MSCs. These differentiated MSCs had MSC characteristics, such as MSC‐specific gene expression and ECM protein secretion. Importantly, EXMCs showed a lower level of inflammatory gene expression and a higher level of anti‐inflammatory gene secretion in contrast to those in UC‐MSCs. Since MSCs are one of the most responsive cell populations to inflammation in tissues, it is worth exploring whether EXMCs have stronger immunomodulatory functions in treating allergic diseases in the future. In addition, inconsistent with UC‐MSCs, E‐MSCs showed the differentiation potential for chondrocytes and osteocytes but lacked the ability to generate adipocytes, indicating that they may be more beneficial for the regeneration of bone tissue cells in orthopaedic diseases. Therefore, whether this type of E‐MSCs can be used to develop a specific cellular drug for treating bone and joint diseases needs to be explored. Thus, the study findings provide a new potential MSC donor for regenerative medicine.

In summary, the present study described a 3D culture method to induce the differentiation of hpESCs into EXMCs by activating the Wnt pathway. The resulting EXMCs exhibited characteristics comparable to those of embryonic EXMCs and n‐EXMCs. Moreover, EXMCs were able to generate significant quantities of ECM proteins and E‐MSCs with the abilities of chondrogenesis and osteogenesis. The study thus provided a protocol to generate EXMCs and verifies their function, suggesting a new idea for EXMC application.

## AUTHOR CONTRIBUTIONS

Tianqing Li conceived and designed the study. Si‐Le Wang and Gao‐Hui Shi performed the cell experiments. Si‐Le Wang and Kui Duan analysed the data. Si‐Le Wang and Yu Yin revised and edited the manuscript. Tianqing Li supervised the project and wrote the manuscript with Si‐Le Wang. All authors read and approved the final version of this manuscript.

## FUNDING INFORMATION

This work was supported by the National Natural Science Foundation of China (32,130,034 and 82,192,874), the National Key Research and Development Program of China (2022YFA1103100 and 2021YFA0805700), the Major Basic Research Project of Natural Science Foundation of Yunnan Province (202102AA100053, 202102AA100007 and YNWR‐YLXZ‐2020‐015) and the Spring City Plan (2022SCP009).

## CONFLICT OF INTEREST STATEMENT

The authors declare no conflict of interest.

## Supporting information


**Data S1.** Figures.

**Table S1.** Antibodies used in the study.
**Table S2.** Primers used in the study.

## Data Availability

The data that supports the findings of this study including RNA‐seq and scRNA‐seq data have not been uploaded.
