# Supplementary material for: Extraembryonic mesoderm cells derived from human embryonic stem cells rely on Wnt pathway activation
Source: Cell Prolif. 2024 Oct 9;58(2):e13761. doi: 10.1111/cpr.13761 (PMC11839190; doi:10.1111/cpr.13761)
Supplement: Supplementary file 1 — Data S1. Figures. [file CPR-58-e13761-s002.docx]

Supplementary Material


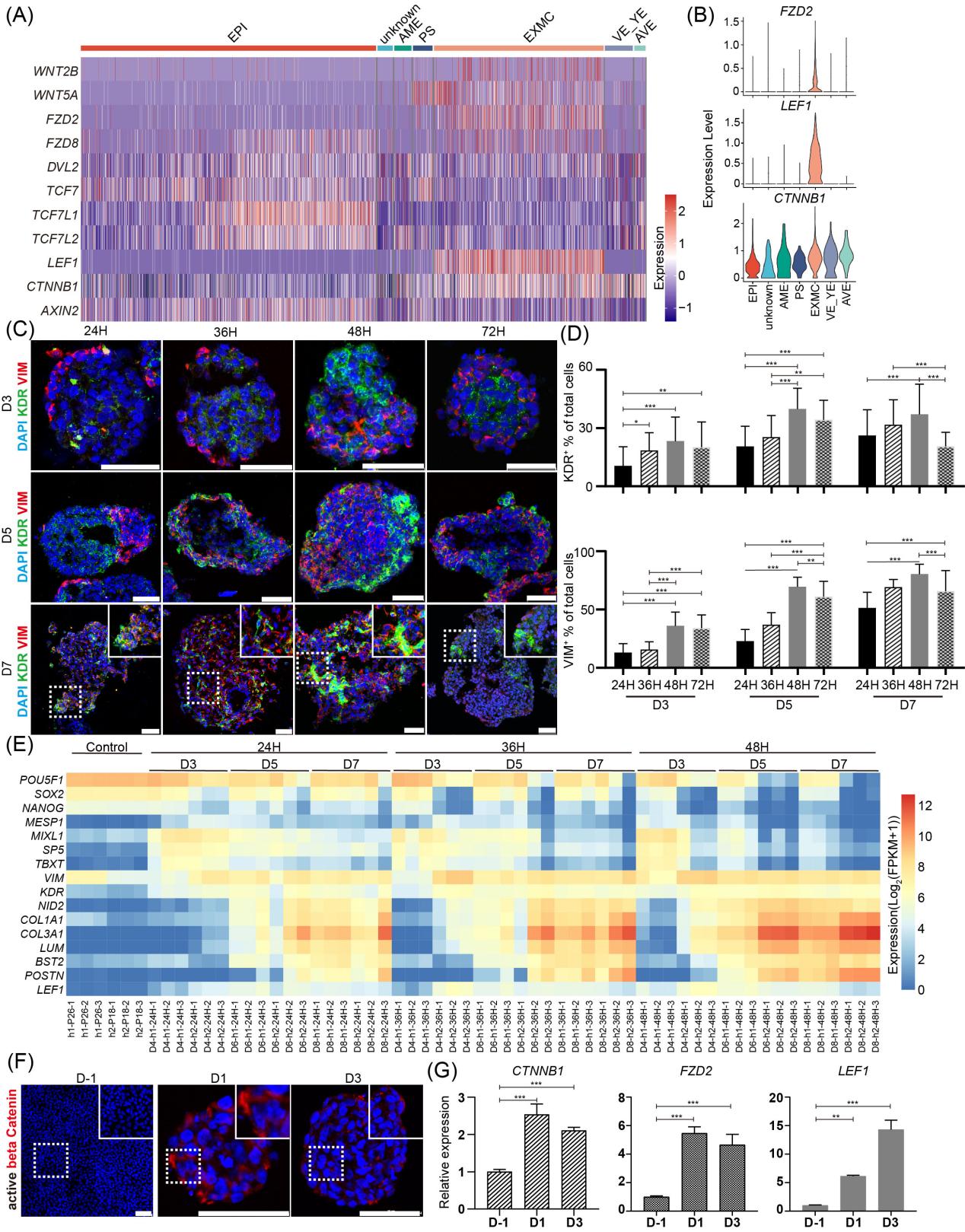


Figure S1. Optimizing culture conditions for differentiation of hpESCs into EXMCs relative to Figure 1. (A) Heatmap of the Wnt pathway-related gene expressions in human post-implantation embryonic lineages. Gene expression levels were normalized. (B) Violin plots for gene expressions in human post-implantation embryonic lineages. (C) Representative immunostaining images of VIM and KDR in EBs treated with CHIR for 24 h (CHIR-24h), 36 h (CHIR-36h), 48 h (CHIR-48h) and 72 h (CHIR-72h) on D3, D5 and D7. Scale bars: 50 μm. (D) Quantification of VIM^+^ and KDR^+^ cell numbers in (C). Data are represented as mean±sd. n=3 in each group. Two-way ANOVA for multiple comparisons was performed. *P<0.05, **P<0.01, and ***P<0.001. (E) Heatmap of gene expressions. EBs treated with CHIR for 24, 36, and 48 h were collected on D3, D5 and D7. hpESCs on D-1 as the control. Gene expression levels were normalized. Results from two hpESC cell lines h1 and h2 are shown, demonstrating no significant differences between them. (F) Representative immunostaining images of active beta Catenin in EBs treated with CHIR for 48 h on D-1, D1 and D3. Scale bars: D-1:100 μm; others: 50 μm.(G) Quantitative gene expression analysis for Wnt downstream genes *CTNNB1* and *LEF1* in EBs treated with CHIR for 48 h on D-1, D1 and D3. Error bars indicate mean±sd. One-way ANOVA for multiple comparisons was performed. *P<0.05, **P<0.01, and ***P<0.001.


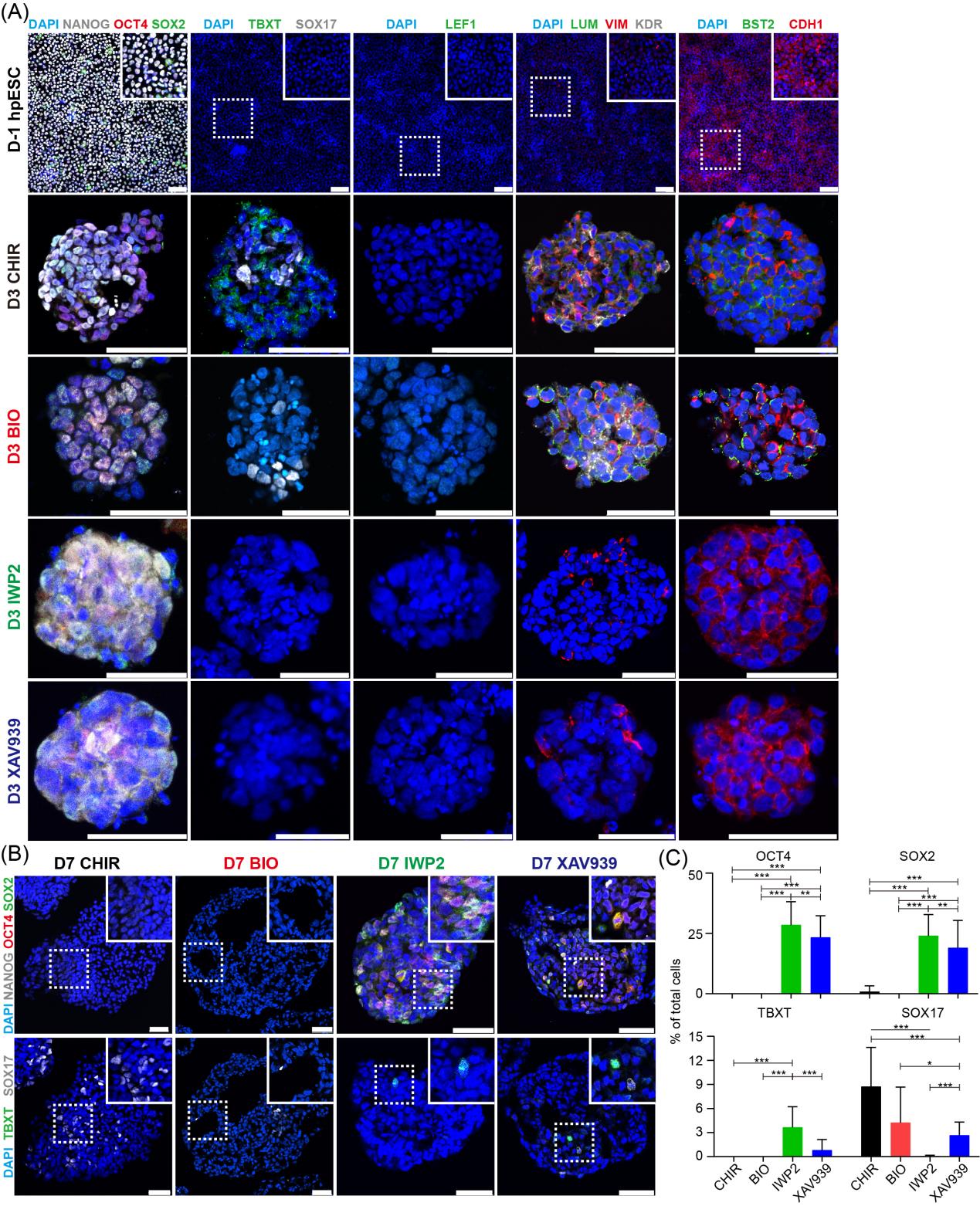


Figure S2. Inhibition of Wnt signaling with IWP2 and XAV939 impeded EXMC differentiation efficiency relative to Figure 2. (A) Representative immunostaining images of markers in hpESCs (D-1) and EBs (D3). Scale bars: 50 μm. (B) Representative immunostaining images of pluripotency (NANOG, SOX2, OCT4), mesoderm (TBXT), and endoderm (SOX17) markers in D7 EBs. Scale bars: 50 μm. (C) Quantification of OCT4^+^, SOX2^+^, TBXT^+^ and SOX17^+^ cells in (B). Data are represented as mean±sd. n=3 in each group. Two-way ANOVA for multiple comparisons was performed. *P<0.05, **P<0.01, and ***P<0.001.


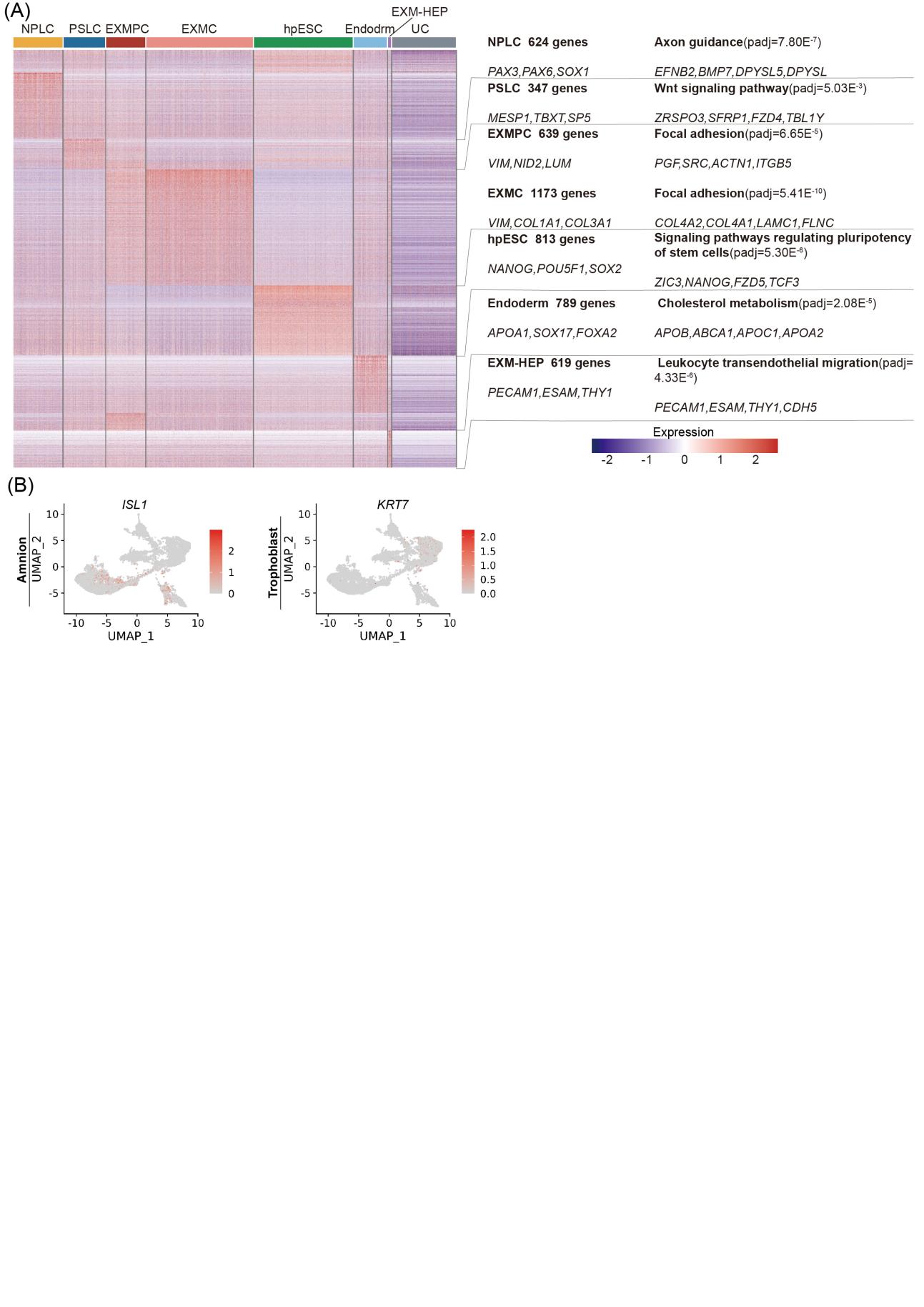


Figure S3. Gene expression profile for EXMCs relative to Figure 3. (A) Heatmap of differentially expressed genes in different cell types shown in Figure 2A. Representative genes (left) and KEGG pathway enrichment analysis (right). (B) UMAP plots of genes expressed in different cell clusters.


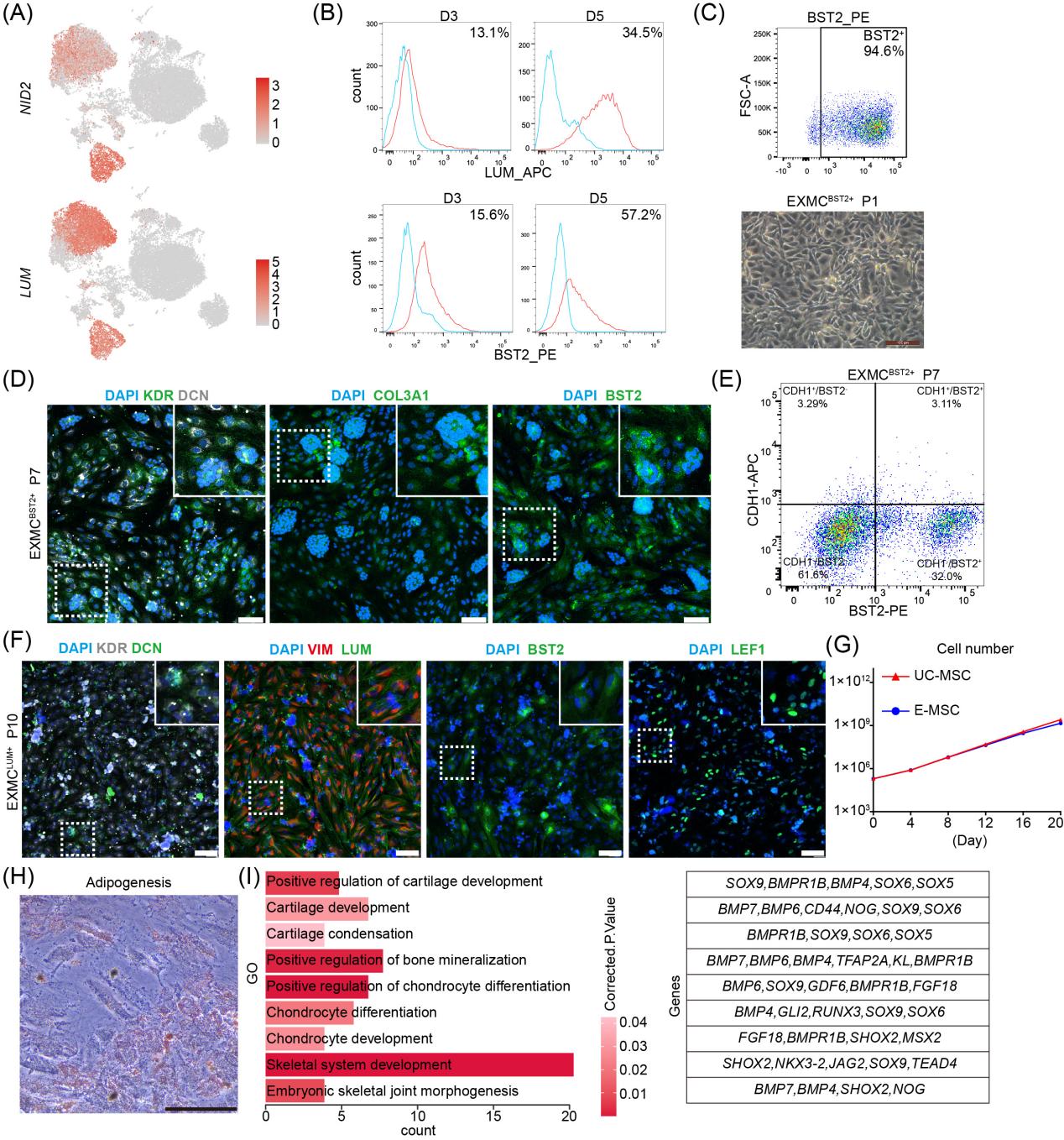


Figure S4. EXMC identification and expansion relative to Figure 5 and Figure 7. (A) UMAP plots of genes expressed in EXMC clusters. (B) Flow cytometry results for LUM^+^ and BST2^+^ cells in D3 and D5 EBs. (C) Flow cytometry data for BST2^+^ cells from D7 EBs (left panel) and morphology of sorted EXMCs^BST2+^ at P1 (right panel). Scale bars: 200 μm. (D) Representative immunostaining images of lineage-specific markers for BST2^+^ cells at P7. Scale bars: 100 μm. (E) Flow cytometry analysis of CDH1 and BST2 markers in BST2^+^ cells at P7. (F) Representative immunostaining images of lineage-specific markers for LUM^+^ cells at P7. Scale bars: 100 μm. (G) Cell proliferation curves for UC-MSCs and E-MSCs. (H) Representative images of E-MSCs after *in vitro* differentiation into adipocytes (Oil Red O). E-MSCs lack adipocyte differentiation. Scale bar: 200 μm. (I) Representative GO terms and genes highly enriched in E-MSCs during osteogenic differentiation compared with UC-MSCs.
