# Supplementary material for: Extraembryonic mesoderm cells derived from human embryonic stem cells rely on Wnt pathway activation
Source: Cell Prolif. 2024 Oct 9;58(2):e13761. doi: 10.1111/cpr.13761 (PMC11839190; doi:10.1111/cpr.13761)
Supplement: Supplementary file 2 — Table S1. Antibodies used in the study. Table S2. Primers used in the study. [file CPR-58-e13761-s001.docx]

| Antibodies | Source | Identifier |
| --- | --- | --- |
| mouse anti-OCT4 | Santa Cruz | cat# SC5279, lot# H1612 |
| rabbit anti-SOX2 | Millipore | cat# AB5603, lot# 2826070 |
| rabbit anti-SOX17 | R&D Systems | cat# 81778, lot# KGA0815042 |
| mouse anti-CDH1 | Abcam | cat# AB76055, lot# GR299147-4 |
| goat anti-NANOG | R&D Systems | cat# AF1997, lot# KKJ0514091 |
| goat anti-TBXT (T) | R&D Systems | cat# AF2085, lot# KQP0617031 |
| rabbit anti-LUM | Abcam | cat# ab168348, lot# 1007676-25 |
| rat IgG1,APC isotype control | Biolegend | cat# 400411, lot# 365119 |
| mouse anti-VIM | eBioscience | cat# 14-9897, lot# 4332786 |
| rabbit anti-LEF1 | Cell signaling technology | cat# 2230S, lot# 8 |
| rabbit anti-DCN | Abcam | cat# ab151988, lot# GR122269-56 |
| rabbit anti-COL3A1 | Abcam | cat# ab7778, lot# GR3441424-5 |
| goat anti-KDR | R&D Systems | cat# AF357, lot# CUE0620061 |
| rabbit anti-GATA4 | Abcam | cat# AB307823, lot# 1043868-3 |
| rabbit anti-BST2 | Abcam | cat#ab243230, lot# 1022489-8 |
| APC anti-mouse CDH1 | Biolegend | cat# 147311, lot# B361314 |
| PE anti-human BST2 | Biolegend | cat# 348405, lot# B373128 |
| mouse IgG1,PE isotype control | Biolegend | cat# 400113, lot# B375705 |
| Mouse anti-active-beta-Catenin | Sigma-Aldrich | cat# 05-665, lot# 3041823 |

Table S1 Antibodies used in the study.

| Gene | Primer sequence (5’ → 3’) |
| --- | --- |
| *Beta-actin*-F | CATGTACGTTGCTATCCAGGC |
| *Beta-actin*-R | CTCCTTAATGTCACGCACGAT |
| *CTNNB1*-F | CATCTACACAGTTTGATGCTGCT |
| *CTNNB1*-R | GCAGTTTTGTCAGTTCAGGGA |
| *LEF1*-F | TGCCAAATATGAATAACGACCCA |
| *LEF1*-R | GAGAAAAGTGCTCGTCACTGT |

Table S2 Primers used in the study.
